# Community Level Correlates of COVID-19 Booster Vaccine Hesitancy in the United States: A Cross-Sectional Analysis

**DOI:** 10.3390/vaccines12020167

**Published:** 2024-02-06

**Authors:** Henry Krasner, Nicolette Harmon, Jeffrey Martin, Crysty-Ann Olaco, Dale M. Netski, Kavita Batra

**Affiliations:** 1 Kirk Kerkorian School of Medicine at UNLV, University of Nevada, Las Vegas, NV 89102, USA; marti167@unlv.nevada.edu (J.M.); olacoc1@unlv.nevada.edu (C.-A.O.); 2 Department of Epidemiology and Biostatistics, School of Public Health, University of Nevada, Las Vegas, NV 89119, USA; harmon2@unlv.nevada.edu; 3 Office of Faculty Affairs, Kirk Kerkorian School of Medicine at UNLV, University of Nevada, Las Vegas, NV 89102, USA; dale.netski@unlv.edu; 4 Department of Medical Education, Kirk Kerkorian School of Medicine at UNLV, University of Nevada, Las Vegas, NV 89102, USA; 5 Office of Research, Kirk Kerkorian School of Medicine at UNLV, University of Nevada, Las Vegas, NV 89102, USA

**Keywords:** COVID-19, vaccine hesitancy, COVID-19 booster dose, community-level intervention, vaccine literacy, functional literacy, communicative literacy, critical literacy, vaccine confidence index

## Abstract

**Introduction**: Evidence exists that individual-level sociodemographic factors contribute to vaccine hesitancy, but it is unknown how community-level factors affect COVID-19 booster dose hesitancy. The current study aims to fill this knowledge gap by comparing data from a nationwide survey on COVID-19 vaccine hesitancy with a community-level indicator, i.e., the Distressed Communities Index (DCI). **Methods**: Attitudes toward vaccinations, vaccine literacy, COVID-19 vaccine confidence index, and trust were measured using a 48-item, psychometrically valid and reliable survey tool. In this study, 2138 survey participants residing in the United States were divided into quintiles of varying community distress levels based on their zip codes using the DCI. Data were analyzed through Chi-square, one-way ANOVA, and post hoc analysis with Tukey’s test. **Results**: A significantly higher proportion of participants from the distressed communities had lower trust than their prosperous counterparts (26.6% vs. 37.6%, *p* < 0.001). On the contrary, participants from the prosperous communities had significantly higher vaccine confidence index scores than those in distressed communities (2.22 ± 1.13 vs. 1.70 ± 1.01, *p* < 0.001). **Conclusions**: These findings affirm the importance of developing community-level interventions to promote trust in COVID-19 vaccinations and increase booster dose uptake. From these results, future studies can examine the efficacy of various community-level interventions.

## 1. Introduction

Since the first reports of a novel Severe Acute Respiratory Syndrome Coronavirus 2 (SARS-CoV-2) infection in 2020, coronavirus-19 (COVID-19) has resulted in 771.8 million confirmed cases and 6.9 million deaths worldwide as of November 2023 [1,2]. During the early stages of the pandemic, the primary two-dose vaccine series against SARS-CoV-2 successfully reduced hospitalizations, reinfection rates, and morbidity associated with COVID-19 infection [3,4]. However, despite the initial success of the primary vaccines, there has been a resurgence in susceptibility to COVID-19 infection in recent years due to viral evolution. This evolution has led to waning immunity against emergent highly divergent strains with the potential of immunity escape of certain highly mutated strains, including Delta in 2020, Omicron in 2021, and Pirola (BA.2.86) and Eris (EG.5) in 2023 [5,6,7,8].

Due to sequence divergence and deteriorating immunity, COVID-19 booster doses have been recommended as a potential measure to reduce the severe clinical outcomes associated with COVID-19 infection. In September 2021, the Food and Drug Administration (FDA) approved booster vaccines, and as of August 2023, 2.79 billion booster doses have been administered worldwide [9,10]. After completion of the primary vaccine series, vaccine effectiveness (VE) against SARS-CoV-2 has been shown to decline to lower than 20% within six months of receiving the second dose [11]. However, in patients who receive the booster dose, VE is maintained at higher levels for extended periods of time [11,12].

Several studies have analyzed the efficacy of the booster dose on a global scale [13,14,15,16]. A study conducted on healthcare workers in Greece found that receiving a booster dose elicits a more robust immune than the immune response achieved after only receiving two doses [14]. In Israel, the booster dose was 93% effective in mitigating severe clinical outcomes, reducing hospitalization and mortality rates [15]. Despite evidence demonstrating the effectiveness of COVID-19 booster doses, global studies have indicated that public hesitancy to an increasing number of COVID-19 vaccinations may threaten vaccine uptake [16].

According to the World Health Organization, vaccine hesitancy is defined as a delay in acceptance or refusal of vaccination despite the availability of vaccination services [1,17]. Concerns regarding initial COVID-19 vaccination doses have also directly translated into a lack of willingness to receive booster shots, limiting the ability to control the pandemic [18]. Despite ample resources, North America and Europe display the highest COVID-19 booster dose hesitancy rates. One study showed that 48.8% of adults who received the primary COVID-19 vaccine series in the United States (U.S.) reported being hesitant to do so [19]. In response, the COVID-19 primary vaccines and booster doses have received exceptional amounts of media attention to promote vaccine uptake [20,21,22,23]. Yet, from 2021 to 2022, booster dose hesitancy has continued to increase globally [24]. Several studies have been conducted to address rising hesitancy rates and to identify the influence of individual-level factors on COVID-19 booster dose acceptance [25,26,27,28,29,30,31,32,33,34,35,36,37,38,39,40]. Some of these studies are outlined in Table 1. Consistently, characteristics such as lower educational attainment, decreased vaccine literacy, and minority racial status were found to be associated with higher levels of vaccine hesitancy on a global scale [27,29,31,35,39,40]. Other factors, including gender, have been shown to play a role in COVID-19 vaccine hesitancy as well, but some of these relationships may vary depending on other variables, such as location [27,31,38].

Current research has thoroughly demonstrated that individual factors play a role in vaccine hesitancy [25,26,27,28,29,31,39,40]. Specific populations, such as those identified as low socioeconomic status, are susceptible to risk factors, including a mistrust in vaccine safety, a lack of education on vaccinations, and inaccessibility to booster doses, all of which may decrease their willingness to receive an additional vaccine [29,30,31,32,35,40]. Vaccine hesitancy may place these marginalized populations at an even higher risk of COVID-19 infection and worse clinical outcomes [33,34,35,36]. To encourage increased COVID-19 booster dose uptake, it is valuable to quantify the variation of booster uptake in different demographic groups to provide insight into which communities are most prone to vaccine hesitancy in the United States.

Despite ample studies demonstrating individual factors associated with hesitancy, there is a lack of knowledge on the role of community-level factors in COVID-19 booster hesitancy. We are filling this knowledge gap by uniquely performing a comprehensive analysis of community-level indicators of socioeconomic status with booster hesitancy. To address the impact of these indicators, we compared data from the 2016–2020 Distressed Community Index (DCI) with a national survey on vaccine hesitancy to investigate the association between socioeconomic status indicators of communities and vaccination acceptance [41,42]. Ultimately, we aim to determine the communities that are most susceptible to COVID-19 booster hesitancy and provide evidence to support the development of resources to mitigate this inequity. In Table 1 below, we identified articles by utilizing key search terms “vaccine hesitancy” and “COVID-19” on a PubMed literature search, followed by individualized article review and selection by researchers.

## 2. Materials and Methods

### 2.1. Study Design

This exploratory, cross-sectional, descriptive study utilizes data from a nationally representative survey. Data were collected from 14 July to 19 July 2021 via Qualtrics (Seattle, WA, USA), a web-based data collection software. Qualtrics is a marketing research service that uses high-quality research panels and quotas to recruit various target populations. Further information regarding the strategy for sampling may be accessed at: https://www.qualtrics.com/research-services/online-sample/ [42]. Qualtrics utilizes panels of participants by acquiring diverse samples of sources through partnerships with numerous online providers. This form of recruitment provides sets of data that adequately represent the desired study population. First, survey participants are randomly chosen by the sample partners via traditional, active, “double-opt-in” market panels, which aim to select respondents who are likely to qualify for studies. In addition, and if needed, social media may be utilized to acquire participants.

### 2.2. Eligibility Criteria (Study Participants and Selection Criteria)

We attempted to recruit a nationally representative sample by gender, race, ethnicity, and region. Qualtrics pooled participants from a variety of sources nationwide to ensure a study sample that represents the current U.S. demography.

Eligibility criteria include current U.S. adults at least 18 years of age who understand English and have the ability to provide voluntary informed consent. Screening questions at the beginning of the survey ensured that the eligibility criteria were met by participants. Participants who did not meet these criteria were excluded from the study. Self-selection and response biases were minimized by hiding details of the survey until the participants successfully met the eligibility requirements. 

### 2.3. Ethical Considerations, Data Privacy, and Quality Control

The Institutional Review Board (IRB) granted this study a category-2 exemption (protocol # 1762717-2). Study participants completed the survey voluntarily and were asked to sign informed consent including all information about the study objective(s), benefits, risks, and potential outcomes. Compensation for completing the survey included incentives, such as airline miles, cash, vouchers, gift cards, charitable donations, and sweepstakes entrance, which were provided per the contract of Qualtrics with their panel providers. 

Qualtrics and the members of the research team adhered to all data privacy laws and regulations. Additionally, the Qualtrics database does not accumulate and save confidential information submitted by respondents. For this study, participant identifiers were removed, and data were then provided to the research team in a spreadsheet protected through password-protected computers. These files were only accessible to research team members.

Various quality features of the survey were utilized to ensure data integrity as well as unique responses from survey participants. These features included digital fingerprinting and monitoring for the prevention of ballot box stuffing to prevent respondents from submitting multiple surveys. Furthermore, participants were excluded from the data set if their total time to complete the survey was significantly faster than the average time of survey respondents due to the potential for a lack of effort in their responses. 

### 2.4. Survey Instrument

The survey component of this study used a 48-item questionnaire that consisted of several psychometrically valid and reliable tools to measure attitudes toward vaccinations generally (9 questions), vaccine literacy (14 questions), COVID-19 vaccine confidence index (8 questions), and demographic information (17 questions). Table 2 describes the survey instrument variables being utilized in this study. The questions regarding general vaccine attitudes were adapted from previous studies that analyzed vaccine hesitancy using a standardized tool for measuring vaccine attitude [43,44]. For the questions regarding vaccine literacy, a self-reported questionnaire was utilized based on the Ishikawa test for chronic, non-communicable disease [45]. The vaccine confidence index (VCI) was adapted from previous studies to inquire about COVID-19 vaccinations specifically. Previously, this VCI was utilized in studies evaluating the influenza vaccine and other vaccine confidence projects globally [44,46]. The instrument variables were calculated as mean scores, especially for vaccine literacy. Values corresponding to each Likert-scale items were summed up. The higher the sum, the higher the literacy. In the case of the vaccine confidence index, there were a total of 8 items, and the score calculation is given below.

1. COVID-19 is a serious illness (A1)
2. COVID-19 vaccine is effective (A2)
3. Healthcare workers must get vaccinated (A3)
4. By getting vaccinated I protect people close to me from COVID-19 (A4)
5. It is better to contract COVID than to get the vaccination (B1)
6. COVID-19 vaccines have serious side effects (B2)
7. COVID-19 Vaccine can cause COVID infection (B3)
8. I am opposed to vaccination (B4)

The vaccine confidence index was calculated as follows:

VCI = [(A1 + A2 + A3 + A4)/4]/[(B1 + B2 + B3 + B4)/4]

**Table 2:** Survey instrument variables.

Construct	Type of Variable	Measurement Scale	Total # of Items	Likert Scale Options
Attitudes towards vaccines	Dependent	Continuous	9	Strongly disagree (1) Disagree (2) Neither agree or disagree (3) Agree (4) Strongly agree (5)
Vaccine Literacy	Dependent	Continuous	14	Never (1) Rarely (2) Sometimes (3) Often (4)
COVID-19 Vaccine Confidence Index	Dependent	Continuous	8	Totally agree (1) Partially agree (2) Partially disagree (3) Totally disagree (4)
Demographics	Independent and Covariates	Continuous and Categorical	17	NA, categorization was performed on an ad hoc basis

Note: Additional variable related to the Distressed Community Index (DCI) was derived from the zip codes of the participants.

### 2.5. Individual and Community-Level Indicators

Multiple individual-level indicators of participant socioeconomic status were analyzed. These factors included race, ethnicity, median household income, education status, rural versus urban residence, pre-existing conditions, and whether there were vulnerable individuals at home [42].

The Distressed Community Index (DCI) is a tool utilized to evaluate and compare the socioeconomic status of communities across the USA by zip code through the analysis of seven economic community-based indicators. These indicators include high school diploma status, housing vacancy rate, adult unemployment, poverty rate, median income ratio, change in employment, and change in establishments. To create these indices, the DCI uses data from the US Census Bureau’s Business Patterns and American Community Survey 5-Year Estimates for 2016–2020. Communities assessed by the DCI include over 99% of the US population. Following assessment, communities are placed into one of five quintiles: prosperous, comfortable, mid-tier, at risk, and distressed. Higher performing quintiles, such as the prosperous quintile, indicate zip codes with the highest levels of economic opportunity. The distressed category indicates zip codes that have been isolated from overall national economic growth. Distressed communities have the highest rates of poverty, housing vacancy, adults without a high school diploma, and “prime age” adults who are not working. They have the lowest median income ratios as well as negative changes in employment and establishments. These groupings of different communities were analyzed in comparison to these communities’ responses to the aforementioned vaccine hesitancy survey to determine the presence of a relationship between the socioeconomic status of communities through the evaluation of individual and community factors with vaccine hesitancy [47].

### 2.6. Statistical Analyses

First, the univariate analysis was performed to describe the data and identify any patterns. Categorical variables were represented as counts and proportions, whereas continuous variables were reported as means and standard deviation unless otherwise stated. The box plot was inspected to assess outliers in the data. The assumption of normality was assessed via Shapiro–Wilk’s test (*p* > 0.05). The assumption of homogeneity of variance was assessed using Levene’s test. Chi-square/Fisher exact and one-way Analysis of Variance (ANOVA) were conducted. The 95% confidence intervals of proportions were calculated by the normal approximation to the binomial calculation. One-way or Welch ANOVA is an omnibus test and only assesses the difference in means among two or more groups. However, it does not provide information on which groups are statistically different. Therefore, Tukey’s post hoc or Games Howell analysis (if assumption of homogeneity of variance was violated) was also performed. The IBM SPSS (V.28) was used to analyze the data, and the level of significance was set at 5%. G*Power software (version 3.1) was used to conduct power analyses [48,49]. Using Cohen’s benchmarks of small effect sizes related to each statistical test (0.1 for Chi-square and 0.1 for one-way ANOVA), alpha level 5%, and 80% power, the maximum sample size required was 1634. After factoring in 20% non-response, the estimated sample size was 1960, comparable to the existing sample size. We used CHAMP guidelines for our statistical reporting [50].

## 3. Results

As shown in Table 3, in a total sample of 2138 survey participants, 61.8% indicated the intent to take a booster dose, while the remaining 38% did not intend to do so. The mean age of the sample was 45.65 ± 18.93 years. The sample was nearly equally split by gender, and over 60% of the sample was white. About 40% of the sample had 4-year college or graduate degree. Over 55% of our sample was from communities ranging from mid-tier or at-risk to the distressed one (Table 3). 

We found a marginal statistically significant difference in the intention of taking a booster dose by community distress level, with a larger proportion of distressed communities not intending to take a booster dose (42.9% vs. 33.8% *p* = 0.05, Table 4). However, some important intersectional axes of individual-level factors and community distress levels were also revealed in Table 4. Among those living in distressed communities, a significantly higher proportion were African Americans (21.3% vs. 6.6%), were single (40.8% vs. 26.5%), had lower educational attainment, were living in South region, and non-metro rural regions as opposed to those belonging to prosperous communities as indicated in Table 4. A significantly higher proportion of participants from the distressed communities had lower trust than their prosperous counterparts (26.6% vs. 37.6%, *p* < 0.001) as indicated in Table 5.

One-way ANOVA was used to compare mean functional literacy, integrative or communicative literacy, and critical literacy scores by community distress level. There was a significant difference in mean scores for functional literacy (*p* = 0.011), integrative or communicative literacy (*p* = 0.009), and critical literacy scores (*p* = 0.004). Post hoc analysis with Tukey’s test showed a significant difference in mean functional literacy scores (*p* = 0.007) and integrative or communicative literacy (*p* = 0.007) between the prosperous and at-risk community levels (Table 6). No other significant differences between groups were observed for these two variables. Post hoc analysis with Tukey’s Test for critical literacy scores showed a significant difference in mean scores between the prosperous and at-risk community levels (*p* = 0.004) and the prosperous and distressed community levels (*p* = 0.019). The one-way Welch ANOVA test was used to compare the mean vaccine confidence index scores by community distress level. There was a significant difference in mean vaccine confidence index scores observed by community distress level (F = 14.793, *p* < 0.001, ω < 0.001). Post hoc analysis with Games–Howell shows a significant difference in average vaccine confidence index scores between prosperous and at-risk communities (*p* < 0.001), prosperous and distressed communities (*p* < 0.001), comfortable and at-risk communities (*p* = 0.040), comfortable and distressed communities (*p* < 0.001), mid-tier and at-risk communities (*p* = 0.044), and mid-tier and distressed communities (*p* < 0.001). As indicated in Table 7, results of the logistic regression indicated that older age, previous primary dose COVID-19 vaccination, living with immunocompromised individuals, and political and regional affiliation were strong predictors of intention to take booster dose. 

## 4. Discussion

The main objective of this study was to identify the communities most susceptible to COVID-19 booster hesitancy by comparing data from a nationwide survey on vaccine hesitancy and the DCI. As SARS-CoV-2 variants continue to mutate, it is increasingly evident that the uptake of COVID-19 booster doses will be vital in further combating the pandemic. This study is the first to examine the intersection of community-level demographic factors with COVID-19 booster dose hesitancy. The major outcomes of this study reflect the variation in COVID-19 information trust, vaccine literacy, and vaccine confidence by community distress level. A higher proportion of participants from the distressed communities had lower trust than their prosperous counterparts. Additionally, participants from prosperous communities had significantly higher vaccine confidence index scores. By elucidating the community-level indicators of COVID-19 booster vaccine hesitancy, interventions can be designed to specifically cater to these populations to promote increased vaccine uptake.

The first significant finding in our study relates to the differences in trust in COVID-19 vaccine information by community distress level, with distressed communities lacking trust in the COVID-19 vaccination. These results corroborate the findings of other studies that discuss the individual-level factors that contribute to COVID-19 vaccine hesitancy in individuals, such as low income, minority racial identity, or decreased education attainment. These data indicate that an increase in the factors that make a community distressed correspond to a higher prevalence of mistrust in COVID-19 vaccine information, which can result in reluctance to receive COVID-19 booster doses. Additionally, reluctance to receive the booster dose may be partly due to a lack of equitable vaccine accessibility in different communities. Studies have thoroughly demonstrated that lower-income populations and more distressed communities are associated with decreased healthcare resource accessibility, including COVID-19 vaccines [51]. Furthermore, this lack of healthcare access may directly correspond to decreased education in vaccination safety and increased medical mistrust, further placing disadvantaged communities in increasingly vulnerable positions. Interestingly, in a global systematic review from 2023, continents associated with higher levels of development and national wealth, such as North America and Europe, were shown to have the highest levels of COVID-19 booster hesitancy [24]. Another study stratifying COVID-19 vaccine uptake by country income status found a seemingly higher willingness to take a COVID-19 vaccine in low- and middle-income countries compared to higher-income countries, such as the United States and Russia [52]. This finding seemingly contrasts our national results describing increased hesitancy in more distressed United States communities but emphasizes that multiple factors influence COVID-19 booster uptake on different scales.

The next significant finding from our results is the relationship between vaccine literacy and vaccine confidence in community distress levels. In all categories surveyed (functional literacy, integrative or communicative literacy, critical literacy, and vaccine confidence index), there was a negative association between scores in these categories and community distress status. For each of these categories, the three most prosperous communities (1, 2, and 3) scored higher than the two lowest quintiles (4 and 5). This corresponds with the previously discussed results, as this suggests that more distressed communities have decreased levels of vaccine literacy and confidence, which may directly play a role in their increased vaccine hesitancy. This may be due to multiple factors, including access to health education and medical mistrust. Studies have shown that in the United States, communities with overall lower income have decreased access to healthcare education, including vaccine safety and efficacy, which may result in a lack of willingness to receive vaccinations [53,54]. On further exploration of the DCI quintiles, we found that the median income ratio of the distressed communities was the lowest, with a range of 18.1–175.2%, as opposed to the prosperous communities with the highest median income ratio of 66.4%, which may extend up to 175.2% (Table 8).

### 4.1. Correlates of Booster Hesitancy

The data characterize the five quintiles by demographic and socioeconomic characteristics that may be associated with COVID-19 booster hesitancy. Among quintiles, race and ethnicity composition of communities were some of the variables found to be significantly different. There was a relationship between White individuals and more prosperous communities, while Black and Hispanic populations were associated with more distressed communities. Minority race status has been thoroughly indicated by other studies to be associated with both lower-income communities and increased vaccine hesitancy, both of which are corroborated by our findings [31,32,39]. A 2021 study demonstrated that Black Americans may be susceptible to many factors that may lead to increased COVID-19 vaccine hesitancy, including significant medical mistrust as a result of systemic racism. This study found that among HIV-positive Black Americans surveyed, nearly all participants endorsed a COVID-19 mistrust belief. Additionally, greater mistrust was associated with greater COVID-19 vaccine and treatment hesitancy [32]. Other studies have further supported these findings, demonstrating that Black and Hispanic sample groups of a survey showed a significantly lower percentage that has received the COVID-19 booster dose compared to White and non-Hispanic Asian populations [31,55,56].

As the prosperity of a community increased, the education level also increased, which has previously been described as playing a role in vaccine hesitancy as well [24,42]. Those with a graduate-level degree or 4-year college degree were highly associated with the most prosperous communities. Another study analyzing sociodemographic variables associated with COVID-19 booster uptake identified that individuals with a doctoral, professional or master’s degree were among groups with the highest proportion of boosted adults in the United States [31]. Increasing levels of education may allow for increased health literacy education opportunities, which has been indicated to promote vaccine uptake. Because race and education level are strongly correlated with vaccine hesitancy, these demographic factors should be targeted for interventions aimed toward promoting COVID-19 booster vaccine uptake.

United States region and county type showed a significant difference between quintiles. It was found that the Southern United States was associated with more distressed quintiles, whereas the Western United States was associated with more prosperous quintiles. A study in 2021 assessing the acceptability of the COVID-19 booster dose in different United States regions similarly found that COVID-19 booster vaccine hesitancy was associated with living in a southern region of the country [42]. In regard to county type, suburban and exurban counties were associated with more prosperous quintiles, while non-metro rural counties were associated with more distress. Other studies about health disparities in rural, urban, and suburban settings corroborate these results. A 2022 study about COVID-19 vaccine hesitancy in rural Midwestern United States towns described that approximately 2/5 of participants surveyed were unwilling to get vaccinated primarily due to knowledge about the vaccine and skepticism about vaccine development and efficacy [57]. Additionally, higher income in suburban and exurban counties generally may allow for increased healthcare infrastructure and disproportionately high accessibility to health resources for residents compared to urban or rural counterparts.

Some findings of this data set require further explanation. For example, political affiliation was found to have no significant relationship with the distress level of communities. However, political affiliation has been shown to be strongly correlated with COVID-19 booster dose and initial vaccination hesitancy [42,58]. Thus, further research is needed to assess the association between political affiliation and vaccination hesitancy within a community-level scope.

### 4.2. Vaccine Literacy and Vaccine Confidence Index

To analyze the relationship between vaccine hesitancy and community distress level, we stratified the quintiles by vaccine literacy and vaccine confidence (Table 4). Previous studies have shown these to be valid predictors of vaccine acceptance, with increased vaccine literacy and confidence associated with increased booster vaccine acceptance [44]. Vaccine literacy is one’s ability to obtain reliable information regarding vaccinations and use this knowledge to make decisions to benefit their health [45]. Vaccine literacy in this analysis consisted of three components: functional, interactive/communicative, and critical vaccine literacy. Functional literacy is competence in reading and writing. Interactive/communicative and critical literacy refer to more advanced skills required to gather meaning in information and use this to make decisions for their own life [42,46]. Meanwhile, vaccine confidence refers to both trust in the safety and efficacy of vaccines in addition to trust in the healthcare system responsible for vaccine administration.

Our data indicated that more prosperous groups were shown to have significantly increased functional, interactive/communicative, and critical vaccine literacy, as well as vaccine confidence. These results corroborate data from other studies. A national United States study found that individuals with lower vaccine literacy were more often low-income, less educated, and more rural [59]. Low-income populations in the United States have been shown to have worse health outcomes and lower life expectancy compared to their higher-income communities, and low vaccine hesitancy in these communities may worsen this present situation, especially as the COVID-19 pandemic continuously evolves over time [60]. As previously mentioned, higher levels of education were inversely related to community distress level (Table 2). It has been shown that increased education is associated with increased COVID-19 vaccine literacy [45]. Higher levels of education may directly allow for increased vaccine literacy and contribute to increased vaccine acceptance in more prosperous communities. Community-based interventions may be useful in improving COVID-19 vaccine literacy in more distressed communities.

### 4.3. Medical Mistrust and Booster Hesitancy

Trust in the medical system, and particularly vaccinations, has been suggested to be an integral contributor to variance in vaccination uptake, including the COVID-19 booster dose. The lack of trust in vaccine information can be attributed to multiple factors, one of which is the spread of misinformation regarding the COVID-19 pandemic and vaccination adverse effects. The World Health Organization coined this widespread vaccine misinformation an “infodemic”, as the public has been supplied with many unfounded scientific claims deterring individuals from receiving vaccination or complying with COVID-19 protocol [61,62]. An American study from 2020 described that individuals who are most susceptible to the COVID-19 infodemic are more likely to have COVID-19 vaccination hesitancy and, unsurprisingly, are less likely to advocate for others to receive vaccination [63]. In another study from the European Union, it was found that trust in science negatively correlated with vaccine hesitancy. However, this study also identified that trust in social media and using social media as a primary news outlet positively correlated with vaccine hesitancy [61]. Addressing the spread of COVID-19 vaccine misinformation will be integral in promoting booster dose uptake on a national scale. In this regard, family physicians treating vulnerable population groups can help in the trust-building process [63].

Beyond the spread of misinformation, other forms of mistrust in the medical system have also dissuaded certain communities from receiving vaccination for COVID-19. As previously mentioned, Black and Hispanic ethnicities were associated with more distressed communities and thus increased COVID-19 booster hesitancy (Table 2 and Table 3). This is unsurprising considering the systemic healthcare factors that promote medical mistrust in these communities. Injustice in health care towards marginalized patient groups has prevented these communities from receiving equitable access to care and led to worse patient outcomes. Regarding COVID-19, one study described that Black patients were significantly less likely to receive antiviral treatment and had longer lengths of in-hospital lengths of stay compared to White patients during the early stages of the pandemic [64]. Another 2022 study looking at incarcerated United States populations specifically identified that COVID-19 mortality and morbidity rates were higher in Black and Hispanic populations compared to their White counterparts [65]. Due to these populations’ association with worse COVID-19 outcomes compared to White populations, increased COVID-19 booster dose hesitancy places them in an even more vulnerable position [36]. It is essential to implement interventions to combat medical mistrust and provide increased COVID-19 booster accessibility to promote vaccine uptake in these communities.

### 4.4. Current State of Interventions

Multiple studies have described, implemented, or proposed interventions designed to combat COVID-19 vaccine hesitancy on a community level. Most of these interventions involve education-based initiatives to enhance vaccine literacy and confidence in individuals. A study in 2022 discussed the development of a digital health resource designed to provide COVID-19 education to young Black adults in the Southern United States to encourage vaccine uptake in this community [66]. Similarly, another study from 2021 proposed to develop a mobile-phone-based intervention in which interactive text messaging could be used to engage specific communities while underscoring the advantages of getting vaccinated against COVID-19 [67]. Another study analyzing COVID-19 vaccine hesitancy among Israeli soldiers discussed the implementation of group consultations regarding initial COVID-19 vaccination and voluntary appointments to discuss their concerns. With the resource implementation, 42.2% of soldiers initially planning not to receive a COVID-19 vaccine ended up receiving the COVID-19 vaccine [68]. Other suggested interventions include engaging religious and community leaders, increasing vaccine accessibility via “pop-up” opportunities to receive vaccination or inquire about COVID-19 vaccine concerns, or implementing sanctions against non-vaccination [69]. There is great strength in taking a community-based approach to developing interventions to promote COVID-19 vaccine uptake. By utilizing these studies as a foundation, more resources can be developed, and current resources can be improved to cater to communities associated with increased COVID-19 booster hesitancy. As the SARS-CoV-2 variants continue to mutate, these interventions will play an increasingly integral role in ensuring protection against COVID-19 and preventing the further spread of infection [70].

Developing and refining interventions such as engaging local leaders, utilizing social media to reach vulnerable populations, integrating educational resources at community centers or schools, and providing additional vaccination opportunities are all possible outlets to advocate for vaccine uptake in distressed communities.

### 4.5. Strengths and Limitations

This study provides valuable insight into community-level factors associated with COVID-19 booster dose hesitancy. A major strength of this study is that it involves a nationally representative cohort. As previously mentioned, to our knowledge, this is the first study conducted analyzing the association between COVID-19 booster vaccine hesitancy and community-level factors.

However, this study additionally has several limitations. We utilized a cross-sectional design, in which data collected displays a specific time frame of attitudes regarding COVID-19 booster vaccine hesitancy. Additionally, opinions about COVID-19 vaccination among different communities are dynamic. By virtue of the cross-sectional design, a cause-and-effect relationship could not be established. Additionally, this study may have a residual confounding bias due to some measures (e.g., role of family physicians) being left unmeasured. Future studies can be planned to account for these factors to gain a holistic view of designing public health interventions.

Next, Qualtrics does not record survey completion or rate of response, meaning that there is no record of the number of individuals invited to participate in the questionnaire. Therefore, we were not able to determine the characteristics of non-respondents. Additionally, web-based surveys may cater to a specific audience, resulting in selection bias impacting the survey participants and potentially limiting generalizability.

## 5. Conclusions

This study demonstrates the results of a national cross-sectional study of the American public regarding COVID-19 booster uptake in different communities stratified by distress level. We present important factors involved in COVID-19 booster hesitancy on a community level, including the demographic factors that compose these different types of communities. There is a significant association between community distress level and vaccine literacy, vaccine confidence, and trust in vaccine information. These components impact the vaccine hesitancy level of communities and are areas to be addressed in designing interventions to ameliorate vaccine inequity. It may be beneficial for future studies to stratify the United States or the global public by different indices to further identify indicators that place specific populations at higher risk of vaccine hesitancy. Given the critical role of booster doses in managing the pandemic, these conclusions are valuable in considering future interventions to increase COVID-19 booster uptake in vulnerable communities.

## Figures and Tables

**Table 1:** Studies examining individual-level factors of COVID-19 vaccine hesitancy.

Study Title	First Author	Year	Aim and Brief Description	Methods	Conclusions and Pertinent Findings
Acceptance, hesitancy and refusal towards COVID-19 vaccination	Raut	2023	Identify determinants that affect COVID-19 vaccine acceptance and hesitancy in hospitalized patients in India	Cross-sectional study of 386 hospital patients in India. Assessed vaccine hesitancy through questionnaire in a one-time interview	- Certain populations displayed higher rates of vaccine hesitancy. These populations included those with low vaccine literacy, those with strong influence from social media and/or family, or those fearing adverse effects. - Higher rates of vaccine acceptance in females than male counterparts
Reasons for COVID-19 vaccine hesitancy in ethnic minority groups: A systematic review and thematic synthesis of initial attitudes in qualitative research	Shearn	2023	Identify common themes related to vaccine hesitancy and barriers to COVID-19 vaccine uptake in racial and ethnic communities and immigrants or refugees during the initial stages of COVID-19 vaccine roll-out (2020–2022)	Systemic literature review of 15 studies across 5 databases (Studies published from December 2021 to February 2022)	Found 3 significant factors that influenced vaccine hesitancy: (1) Institutional mistrust, (2) Lack of confidence in the efficacy of the vaccine and safety/adverse effects or the speed of vaccine development and roll out, (3) Difficulty accessing trustworthy information or access to misinformation
A Systematic Review on Sociodemographic, Financial and Psychological Factors Associated with COVID-19 Vaccine Booster Hesitancy among Adult Population	Ayyalasomayajula	2023	Defined major determinants of vaccine hesitancy in adult populations	Systematic review of articles pooled from several databases (Scopus, PubMed, Embase). These studies investigated factors related to vaccine hesitancy on a global scale. All studies were published between January 2021 and October 2022.	Multifactorial nature to vaccine hesitancy; major factors of hesitancy are categorized into 3 groups (sociodemographic, financial, psychological groups) and based on individual factors (such as the influence of social media or the government).
Knowledge, Attitudes, and Practices of Adult Iraqi Population Towards COVID-19 Booster Dose: A Cross-Sectional Study	Al-Qerem	2022	Evaluation of knowledge, attitudes, and practices towards COVID-19 booster doses in adults living in Iraq	Cross-sectional survey to analyze attitudes to the booster vaccine and individual factors that may be associated with these attitudes	High rates of vaccine hesitancy in Iraqi adults who are either low socioeconomic status and/or have limited knowledge of COVID-19 vaccines and their efficacy
Geographic, Occupational, and Sociodemographic Variations in Uptake of COVID-19 Booster Doses Among Fully Vaccinated US Adults, 1 December 2021, to 10 January 2022	Agaku	2022	Examine the variation in COVID-19 booster dose hesitancy among fully vaccinated U.S. adults from different geographic locations, occupation, and sociodemographic categories	Cross-sectional survey with data from an online platform, Household Pulse Survey, conducted by the U.S. Census Bureau	In the U.S., less than half of adults who were fully vaccinated against COVID-19 received the booster dose. Vaccine hesitancy is multi-factorial, but higher booster vaccine uptake was demonstrated in males, older populations, and those with higher education.
Parents’ willingness to vaccinate themselves and their children with the booster vaccine against SARS-CoV-2: A cross-sectional study in Puyang City, China	Zhou	2022	Examines hesitancy/willingness of parents to vaccinate themselves and their children with a booster dose against COVID-19	Cross-sectional study with a sample of parents of children (children aged 3–17) in China, questionnaire on Wen-Juan Xing platform (online survey)	- 2/5 of participants who were eligible to receive the booster dose of COVID-19 vaccine exhibited vaccine hesitancy - Effectiveness and safety of vaccines are the main factors that influence vaccine acceptance
Racial And Ethnic Disparities In COVID-19 Booster Uptake	Baker	2022	Assess racial and ethnic disparities in vaccine uptake	Used data from the CDC to compare rates of the first COVID-19 vaccine dose and the first booster dose among several ethnic groups (Asian, Black, Hispanic, White) in the United States	Booster uptake rates were higher among Asian and White populations and lowest in Black and Hispanic populations
COVID-19 Vaccine Hesitancy and Its Associated Factors in Japan	Okubo	2021	Analyze vaccine hesitancy rates in the Japanese population using a larger sample and to review individual-level factors that may be associated with vaccine hesitancy.	Cross-sectional survey online using the Japan “COVID-19 and Society” Internet Survey (JACSIS) for adults living in Japan	- Several factors were implicated in an individual’s willingness to receive the COVID-19 booster doses. Such factors included age, gender, and safety concerns - 70% of participants cited concerns regarding the safety of the vaccine as the main reason for their hesitancy
Socioeconomic disparities and COVID-19 vaccination acceptance: a nationwide ecologic study	Caspi	2021	Assess the relationship between COVID-19 vaccination rates and individuals’ socioeconomic status	Ecological study with data from the Israel Ministry of Health	Regions with lower SES have a higher disease burden but are less likely to receive the COVID-19 vaccine
Predictors of Vaccine Hesitancy: Implications for COVID-19 Public Health Messaging	Hudson	2021	To identify the demographic or individual differences that may contribute to vaccine hesitancy to inform public health campaigns	Literature review of studies spanning from 2006 to 2023 assessing the relationship between vaccine attitudes and individual-level factors	Individual factors affecting vaccine acceptance/hesitancy include age, socioeconomic status, education and health literacy, parental status, rurality, mistrust in authority, disgust sensitivity, and risk aversion

**Table 3:** Characteristics of sample used in this study (N = 2138).

Variable Name	Categories	N (%)	95% CI
Intention to take booster dose	Yes	1322 (61.8)	[59.7; 63.9]
	No	816 (38.2)	[36.1; 40.3]
Age (Mean ± SD) in years	-	45.65 ± 18.93	[44.9; 46.5]
Gender	Female	1063 (49.7)	[47.6; 51.8]
	Male	1018 (47.6)	[45.5; 49.7]
Race/ethnicity	White	1355 (63.4)	[61.4; 65.4]
	African American	277 (13.0)	[11.6; 14.4]
	Hispanic	333 (15.6)	[14.1; 17.1]
	Other (including multiracial groups)	173 (8.1)	[6.9; 9.3]
Marital status	Divorced/Separated/Widowed	472 (22.1)	[20.3; 23.9]
	Married	945 (44.2)	[42.1; 46.3]
	Single, never married	721 (33.7)	[31.7; 35.7]
Education	High school diploma or GED	515 (24.1)	[22.3; 25.9]
	4-year college degree	482 (22.5)	[20.7; 24.3]
	Graduate level degree	356 (16.7)	[15.1; 18.3]
	Some college	666 (31.2)	[29.2; 33.2]
	Some high school	93 (4.3)	[3.4; 5.2]
	Other	26 (1.2)	[0.7; 1.7]
Health insurance	Yes	1879 (87.9)	[86.5; 89.3]
	No	222 (10.4)	[9.1; 11.7]
Region	Midwest	481 (22.5)	[20.7; 24.3]
	Northeast	404 (18.9)	[17.2; 20.6]
	South	774 (36.2)	[34.2; 38.2]
	West	479 (22.4)	[20.6; 24.2]
Political affiliation	Democrat	830 (38.8)	[36.7; 40.9]
	Republican	573 (26.8)	[24.9; 28.7]
	Independent	586 (27.4)	[25.5; 29.3]
	Other	25 (1.2)	[0.7; 1.7]
Religion	Roman Catholic	485 (22.7)	[20.9; 24.5]
	Protestant	478 (22.4)	[20.6; 24.2]
	Religiously unaffiliated	652 (30.5)	[28.6; 32.5]
	Other	523 (24.5)	[22.7; 26.3]
Distressed community index quintiles	Prosperous	452 (21.1)	[19.4; 22.8]
	Comfortable	433 (20.3)	[18.6; 22.0]
	Mid-tier	395 (18.5)	[16.9; 20.1]
	At risk	423 (19.8)	[18.1; 21.5]
	Distressed	380 (17.8)	[16.2; 19.4]
County type	Large urban	749 (35.0)	[33.0; 37.0]
	Small urban	319 (14.9)	[13.4; 16.4]
	Suburban	269 (12.6)	[11.2; 14.0]
	Exurban	234 (10.9)	[9.6; 12.2]
	Metro rural	242 (11.3)	[10.0; 12.6]
	Non-metro rural	270 (12.6)	[11.2; 14.0]

**Table 4:** Comparing sample characteristics by community distress level (N = 2138).

Variable Name	Categories	1	2	3	4	5	*p* Value	Statistics	ES
Intention to take booster dose	Yes	299 (66.2)	270 (62.4)	250 (63.3)	249 (58.9)	217 (57.1)	0.05	9.123	0.066
	No	153 (33.8)	163 (37.6)	145 (36.7)	174 (41.1)	163 (42.9)			
Intention to have children vaccinated/ boosted	Yes	88 (51.8)	86 (46.2)	83 (49.1)	100 (46.5)	91 (43.1)	0.528	3.181	0.058
	No/Not sure	82 (48.2)	100 (53.8)	86 (50.9)	115 (53.5)	120 (56.9)			
Age (Mean ± SD)	-	49.74 ± 19.79	47.10 ± 19.58	47.27 ± 18.98	44.13 ± 17.97	40.24 ± 16.44	<0.001	0.027	0.029
Gender	Female	225 (50.8)	202 (47.9)	192 (49.6)	224 (54.0)	200 (55.6)	0.181	6.258	0.056
	Male	218 (49.2)	220 (52.1)	195 (50.4)	191 (46.0)	160 (44.4)			
Race/ethnicity	White	315 (69.7)	302 (69.7)	250 (63.3)	246 (58.2)	210 (55.3)	<0.001	71.812	0.107
	African American	30 (6.6)	49 (11.3)	45 (11.4)	67 (15.8)	81 (21.3)			
	Hispanic	62 (13.7)	48 (11.1)	60 (15.2)	80 (18.9)	71 (18.7)			
	Other (including multiracial groups)	45 (10.0)	34 (7.9)	40 (10.1)	30 (7.1)	18 (4.7)			
Marital status	Divorced/Separated/Widowed	95 (21.0)	84 (19.4)	92 (23.3)	108 (25.5)	85 (22.4)	<0.001	33.383	0.090
	Married	237 (52.4)	209 (48.3)	161 (40.8)	170 (40.2)	140 (36.8)			
	Single, never married	120 (26.5)	140 (32.3)	142 (35.9)	145 (34.3)	155 (40.8)			
Education	High school diploma or GED	73 (16.2)	95 (21.9)	95 (24.1)	118 (27.9)	122 (32.1)	<0.001	82.155	0.099
	4-year college degree	122 (27.0)	108 (24.9)	84 (21.3)	82 (19.4)	79 (20.8)			
	Graduate level degree	103 (22.8)	80 (18.5)	54 (13.7)	59 (13.9)	39 (10.3)			
	Some college	144 (31.9)	128 (29.6)	138 (34.9)	135 (31.9)	107 (28.2)			
	Some high school	8 (1.8)	15 (3.5)	18 (4.6)	26 (6.1)	26 (6.8)			
	Other	2 (0.4)	7 (1.6)	6 (1.5)	3 (0.7)	7 (1.8)			
Health insurance	Yes	406 (90.8)	384 (89.7)	351 (90.5)	362 (87.4)	328 (87.9)	0.413	3.952	0.044
	No	41 (9.2)	44 (10.3)	37 (9.5)	52 (12.6)	45 (12.1)			
Friends/Family tested positive for COVID-19	Yes	217 (48.4)	210 (48.7)	190 (49.0)	195 (47.1)	186 (49.9)	0.959	0.640	0.018
	No	231 (51.6)	221 (51.3)	198 (51.0)	219 (52.9)	187 (50.1)			
Living with vulnerable/ immuno- compromised person	Yes	146 (32.8)	146 (34.3)	124 (32.7)	139 (33.9)	124 (33.2)	0.986	0.351	0.013
	No	299 (67.2)	280 (65.7)	255 (67.3)	271 (66.1)	250 (66.8)			
Pre-existing conditions	Yes	192 (43.3)	188 (44.2)	187 (48.6)	203 (49.5)	152 (41.6)	0.115	7.422	0.060
	No	251 (56.7)	237 (55.8)	198 (51.4)	207 (50.5)	213 (58.4)			
Region	Midwest	104 (23.0)	96 (22.2)	83 (21.0)	96 (22.7)	89 (23.4)	<0.001	45.573	0.085
	Northeast	86 (19.0)	91 (21.0)	73 (18.5)	88 (20.8)	55 (14.5)			
	South	142 (31.4)	137 (31.6)	140 (35.4)	157 (37.1)	182 (47.9)			
	West	120 (26.5)	109 (25.2)	99 (25.1)	82 (19.4)	54 (14.2)			
Political affiliation	Democrat	170 (39.3)	177 (42.9)	142 (38.4)	163 (41.6)	154 (43.4)	0.438	12.097	0.045
	Republican	124 (28.6)	126 (30.5)	118 (31.9)	108 (27.6)	86 (24.2)			
	Independent	134 (30.9)	108 (26.2)	106 (28.6)	115 (29.3)	108 (30.4)			
	Other	5 (1.2)	2 (0.5)	4 (1.1)	6 (1.5)	7 (2.0)			
Religion	Roman Catholic	120 (26.5)	97 (22.4)	91 (23.0)	79 (18.7)	79 (20.8)	0.010	26.329	0.065
	Protestant	123 (27.2)	99 (22.9)	85 (21.5)	89 (21.0)	75 (19.7)			
	Religiously unaffiliated	119 (26.3)	128 (29.6)	124 (31.4)	130 (30.7)	132 (34.7)			
	Other	90 (19.9)	109 (25.2)	95 (24.1)	125 (29.6)	94 (24.7)			
County type	Large urban	132 (29.2)	160 (37.0)	151 (38.2)	169 (40.0)	137 (36.1)	<0.001	171.427	0.143
	Small urban	79 (17.5)	69 (15.9)	55 (13.9)	52 (12.3)	64 (16.8)			
	Suburban	107 (23.7)	72 (16.6)	39 (9.9)	27 (6.4)	24 (6.3)			
	Exurban	70 (15.5)	49 (11.3)	44 (11.1)	45 (10.6)	26 (6.8)			
	Metro rural	45 (10.0)	45 (10.4)	55 (13.9)	47 (11.1)	50 (13.2)			
	Non-metro rural	19 (4.2)	38 (8.8)	51 (12.9)	83 (19.6)	79 (20.8)			

Community distress levels are categorized into five levels: 1 = prosperous, 2 = comfortable, 3 = mid-tier, 4 = at risk, and 5 = distressed.

**Table 5:** Differences in the trust in COVID-19 vaccine information by community distress level.

Variable Name	Categories	1	2	3	4	5	*p* Value	Statistics	ES
How much trust in COVID-19 vaccine information, n (%)	Not at all	36 (8.0)	53 (12.2)	37 (9.4)	60 (14.2)	48 (12.6)	<0.001	33.015	0.073
	Very little	71 (15.7)	78 (18.0)	76 (19.2)	93 (22.0)	87 (22.9)			
	Somewhat	175 (38.7)	152 (35.1)	156 (39.5)	162 (38.3)	144 (37.9)			
	A lot	170 (37.6)	150 (34.6)	126 (31.9)	108 (25.5)	101 (26.6)			

Community distress levels are categorized into five levels: 1 = prosperous, 2 = comfortable, 3 = mid-tier, 4 = at risk, and 5 = distressed. ES = effect size. Effect size is reported as Cramer’s V coefficient.

**Table 6:** Vaccine literacy and vaccine confidence index by community distress level.

Variable Name	1	2	3	4	5	*p* Value
Functional literacy	3.05 ± 0.69	2.98 ± 0.74	2.96 ± 0.77	2.88 ± 0.74	2.91 ± 0.78	0.011
Integrative or communicative literacy	3.10 ± 0.62	3.04 ± 0.69	3.01 ± 0.67	2.95 ± 0.65	2.98 ± 0.66	0.009
Critical literacy	3.19 ± 0.70	3.11 ± 0.75	3.09 ± 0.72	3.02 ± 0.71	3.04 ± 0.72	0.004
Vaccine confidence index	2.22 ± 1.13	2.02 ± 1.14	2.02 ± 1.12	1.81 ± 1.11	1.70 ± 1.01	<0.001

Community distress levels are categorized into five levels: 1 = prosperous, 2 = comfortable, 3 = mid-tier, 4 = at risk, and 5 = distressed.

**Table 7:** Logistic Regression Predicting the likelihood of intention to take booster dose by selected independent variables (N = 2138).

Categories	Odds Ratio	95% Confidence Limits		*p* Value
		Lower	Upper	
Age	1.023	1.012	1.035	**<0.0001**
Vaccinated with primary dose yes vs. no (ref.)	7.570	5.436	10.541	**<0.0001**
Male vs. Female (ref.)	1.211	0.887	1.653	0.2284
Hispanic vs. Black (ref.)	0.562	0.300	1.053	0.0721
White vs. Black (ref.)	0.702	0.426	1.158	0.1656
Other vs. Black (ref.)	0.801	0.383	1.672	0.5540
Married vs. Divorced/separated/widowed (ref.)	1.146	0.759	1.731	0.5161
Single vs. Divorced/separated/widowed (ref.)	0.891	0.568	1.399	0.6173
Graduate level degree vs. Some high school (ref.)	1.435	0.617	3.337	0.4016
Four-year college degree vs. Some high school (ref.)	1.185	0.541	2.600	0.6712
High school diploma or GED vs. Some high school (ref.)	0.849	0.404	1.785	0.6668
Some college vs. Some high school (ref.)	0.823	0.393	1.723	0.6047
Other vs. Some high school (ref.)	1.002	0.174	5.756	0.9985
Distress community score	1.001	0.995	1.006	0.7898
Income ≤ USD 10,000 (Ref. USD 250,001+)	0.667	0.187	2.373	0.5313
USD 10,001 to USD 25,000 (Ref. USD 250,001+)	0.545	0.160	1.860	0.3324
USD 25,001 to USD 50,000 (Ref. USD 250,001+)	0.491	0.149	1.625	0.2444
USD 50,001 to USD 100,000 (Ref. USD 250,001+)	0.507	0.155	1.659	0.2617
USD 100,001 to USD 250,000 (Ref. USD 250,001+)	0.763	0.230	2.526	0.6577
Health insurance: Yes vs. No (ref.)	1.267	0.746	2.153	0.3809
Infected with COVID-19: Yes vs. No (ref.)	1.217	0.892	1.659	0.2157
Living with vulnerable group: Yes vs. No (ref.)	1.633	1.141	2.339	**0.0074**
Pre-existing disorders: Yes vs. No (ref.)	1.052	0.752	1.471	0.7689
Midwest region vs. South (ref.)	0.962	0.666	1.390	0.8365
Northeast region vs. South (ref.)	1.055	0.717	1.553	0.7840
West region vs. South (ref.)	0.202	0.047	0.868	**0.0315**
Independent political affiliation vs. Republican (ref.)	0.950	0.640	1.409	0.7974
Democrat vs. Republican (ref.)	2.210	1.486	3.285	**<0.0001**
Other vs. Republican (ref.)	0.949	0.287	3.137	0.9320
Roman Catholic vs. Protestant (ref.)	1.508	0.962	2.366	0.0735
Other religions vs. Protestant (ref.)	1.699	1.084	2.665	**0.0209**
No religion preference vs. Protestant (ref.)	1.279	0.836	1.956	0.2567

Note: *p* values bolded in the table are statistically significant.

**Table 8:** Median income ratios corresponding to distress quintiles.

Distress Quintile	Median Income Ratio
1 [Prosperous]	66.4–369%
2 [Comfortable]	46.6–367.7%
3 [Mid-tier]	31.1–316.7%
4 [At risk]	26.2–218.9%
5 [Distressed]	18.1–175.2%

## Data Availability

The data presented in this study are available upon request from the corresponding author. The data are not publicly available due to ethical reasons.
